# Photo-stability study of a solution-processed small molecule solar cell system: correlation between molecular conformation and degradation

**DOI:** 10.1080/14686996.2018.1433948

**Published:** 2018-02-22

**Authors:** Michael J. Newman, Emily M. Speller, Jérémy Barbé, Joel Luke, Meng Li, Zhe Li, Zhao-Kui Wang, Sagar M. Jain, Ji-Seon Kim, Harrison Ka Hin Lee, Wing Chung Tsoi

**Affiliations:** ^a^ SPECIFIC, Department of Engineering, Swansea University, Swansea, UK; ^b^ Department of Physics and Centre for Plastic Electronics, Imperial College London, London, UK; ^c^ Institute of Functional Nano & Soft Materials (FUNSOM), Soochow University, Suzhou, China

**Keywords:** Small molecule solar cells, BTR, photobleaching, burn-in, Raman spectroscopy, molecular conformation, 50 Energy Materials, 101 Self-assembly / Self-organized materials, 209 Solar cell / Photovoltaics, 302 Crystallization / Heat treatment / Crystal growth, 505 Optical / Molecular spectroscopy

## Abstract

Solution-processed organic small molecule solar cells (SMSCs) have achieved efficiency over 11%. However, very few studies have focused on their stability under illumination and the origin of the degradation during the so-called burn-in period. Here, we studied the burn-in period of a solution-processed SMSC using benzodithiophene terthiophene rhodamine:[6,6]-phenyl C_71_ butyric acid methyl ester (BTR:PC_71_BM) with increasing solvent vapour annealing time applied to the active layer, controlling the crystallisation of the BTR phase. We find that the burn-in behaviour is strongly correlated to the crystallinity of BTR. To look at the possible degradation mechanisms, we studied the fresh and photo-aged blend films with grazing incidence X-ray diffraction, UV–vis absorbance, Raman spectroscopy and photoluminescence (PL) spectroscopy. Although the crystallinity of BTR affects the performance drop during the burn-in period, the degradation is found not to originate from the crystallinity changes of the BTR phase, but correlates with changes in molecular conformation – rotation of the thiophene side chains, as resolved by Raman spectroscopy which could be correlated to slight photobleaching and changes in PL spectra.

## Graphical abstract

**Figure:**
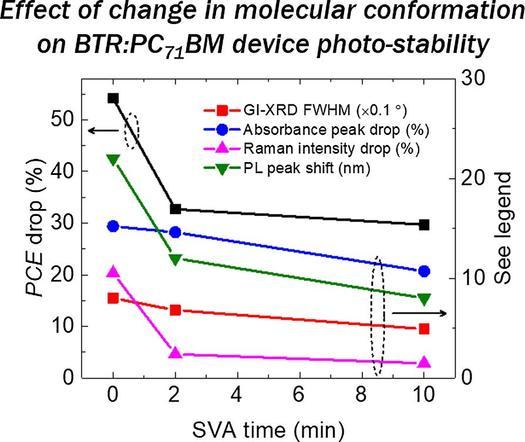

## 1. Introduction

Solution-processed organic small molecule solar cells (SMSCs) have a number of advantages over the more common polymer solar cells (PSCs), including well-defined chemical structure and thus monodisperse molecular weight, which allows easier purification and better reproducibility [1]. Power conversion efficiency (*PCE*) over 11% has been recently achieved [2,3], which is comparable to most of the state-of-the-art PSCs [4]. However, the stability of solution-processed SMSCs is far less understood than PSCs, even though stability remains a critical consideration for their commercialisation [5]. Although many researchers have studied the stability of SMSCs with evaporated active materials [6–9], there are only a few reports focusing on the stability of SMSCs with solution-processed active layers [10,11]. Apparently, there are several fundamental differences between both types of small molecules such as the planarity of the molecules or the inclusion of side chains on the molecule leading to the difference in their solubility. Besides, in term of devices, the device architecture of SMSCs are usually different, bilayer structure is employed for most evaporated SMSCs and bulk-heterojunction structure with solvent vapour annealing (SVA) treatment for most solution-processed SMSCs [1,12].

Recently, solution-processed SMSCs using BTR:PC_71_BM have attracted a lot of attention for its promising efficiency of 9.3%, and high fill factor (*FF*) of 77% with optimal active layer thickness of over 200 nm [13]. Even with the thickness of up to 400 nm, which is more preferable for up-scaling, the efficiency can still be as high as 8% [13]. To achieve high efficiency for most of the solution-processed SMSC systems including BTR:PC_71_BM, SVA is a common method for the optimisation [1], which can increase/control the degree of crystallisation of the small molecule donor in the active layers [13]. Besides, it has been demonstrated that mixing BTR with a polymer:fullerene system, forming a ternary blend, can boost the *PCE* as well as increase the optimal active layer thickness [14].

Burn-in is a widely observed phenomenon occurring at the beginning of the degradation under light and inert environment for polymer/small molecule organic solar cells, in which the device efficiency drops exponentially [15–17]. Overcoming the burn-in effect is a general but critical challenge for organic solar cells since the drop in efficiency can be up to 50–60% [10,18], where the burn-in period can be from hundreds of hours to over a thousand hours [18]. However, the origins of the burn-in effect are still widely debated [19–28]. Besides, the degradation mechanism of solution-processed SMSCs could be different from evaporated SMSCs and PSCs due to their intrinsic molecular difference and the variation of processing methods. Here, we attempt to understand the degradation during the burn-in period of BTR:PC_71_BM devices with different levels of crystallinity of the active layers by controlling the SVA time, through studying the structural and optical properties of the photo-aged films. To minimise the stress factors, both the devices and films are degraded at room temperature under irradiation from visible light-emitting diodes (LEDs) with a constant flow of dry nitrogen for up to 192 h (8 days).

## 2. Experimental

BTR and PC_71_BM were purchased from 1-Material and Solenne BV, respectively. Chloroform and tetrahydrofuran (THF) were purchased from Sigma-Aldrich (Gillingham, UK). Poly(3,4-ethylenedioxythiophene)-poly(styrenesulfonate) (PEDOT:PSS) was purchased from Heraeus (Clevios P VP AI 4083; Heraeus Conductive Polymers (Europe), Leverkusen, Germany). All materials were used as received.

Glass substrates covered with indium tin oxide (ITO, 15 Ω/□) were cleaned sequentially with detergent, deionised water, acetone and isopropyl alcohol in an ultrasonic bath. BTR and PC_71_BM (1:1 weight ratio) were dissolved and stirred in chloroform with a total concentration of 40 mg/ml at 60 °C in a nitrogen-filled glovebox. PEDOT:PSS was spin-coated onto plasma-cleaned ITO substrates in air followed by 150 °C annealing for 10 min. The blend solution was then spin-coated onto the PEDOT:PSS-coated substrates at an optimised speed of 1500 r.p.m. for 15 s in the nitrogen-filled glovebox resulting in active layer thickness of 220 nm as measured by a profilometer. SVA treatment was performed in a sectioned petri dish with 1 ml THF filled in a section of the petri dish. The petri dish was covered with a lid for at least 2 min before performing SVA to the active layer. The samples were placed in the other sections of the petri dish for different exposure times as mentioned in the main text. Then, 30 nm of calcium and 100 nm of aluminium were evaporated onto the active layer in an evaporator with base pressure of 2 × 10^−5^ mbar, forming devices with active area of 0.15 cm^2^. The devices were encapsulated with glass slides using epoxy before the measurements in air. *J*-*V* scans were performed by a sourcemeter (Keithley 2400; Tektronix Inc., Bracknell, UK) under a solar simulator (Newport 92193A-1000; Newport Spectra-Physics Ltd., Didcot, UK) with intensity of 90 mW/cm^2^.

Device burn-in measurements were performed with a home-built electrical environmental chamber filled with dry nitrogen at a controlled temperature of around 25 °C, under constant illumination of one-sun equivalent white LEDs arrays. The intensity of the LEDs was adjusted to one-sun equivalent so that the initial *J*_*SC*_ of the devices measured in the chamber were matched with the *J*_*SC*_ measured under one-sun illumination. A sourcemeter was used to obtain the *J*-*V* data during the degradation for every 15 min. BTR:PC_71_BM blend films were prepared on quartz substrates for the grazing-incidence X-ray diffraction (GI-XRD), UV–vis absorbance, Raman and photoluminescence (PL) measurements, using the same preparation of the active layers of the devices. These films were degraded in the same way as the device degradation stated above to ensure the relevance to the device stability studies.

GI-XRD measurements were carried out using a Bruker D8 Discover instrument (Bruker AXS LTD., Coventry, UK) with a CuK α beam (wavelength = 0.15418 nm), in the range from 1° to 10° with scan parameters of 2 s/step, 0.02° step size and fixed incidence angle of 1°.

The UV–vis absorbance spectra were measured with a Perkin Elmer Lambda 750 spectrophotometer (PerkinElmer, Seer Green, UK), using a quartz substrate as a reference sample for calibration.

For the Raman and PL measurements, the blend films were measured under a dry nitrogen environment which was enabled by purging an environmental chamber (Linkam THMS600; Linkam Scientific Instruments, Tadworth, UK) with dry nitrogen for ~5 min prior to the measurements, and maintaining a positive pressure during measurements. The Raman and PL measurements were performed with a Renishaw inVia Raman system (Renishaw plc., Wotton-Under-Edge, UK) in backscattering configuration. A 532 nm laser and 50x objective were used (NA: 0.50, spot size ≈ 1 μm). For the micro-Raman measurements, a laser power of 0.03 mW and acquisition time of 60 s was used. For the micro-PL measurements, a laser power of 0.03 mW and acquisition time of 0.01 s was used. 1800 l/mm and 300 l/mm gratings were used for the Raman and PL measurements, respectively.

Density function theory (DFT) simulations were carried out using GAUSSIAN09 [29] on the Imperial College High-Performance Computing Service. All DFT simulations were carried out at the B3LYP level of theory, using the basis set 6311G(d,p) [30–33]. All alkyl side chains were simplified to methyl groups to reduce computational time. The structure of BTR was optimised to an energy minimum and compared to other conformations to find the global minimum energy structure. Frequency calculations were carried out to simulate the Raman spectra, an empirical scaling factor of 0.9669 was used for the frequency of vibration [34]. The frequency modes calculated were visualised using the GAUSSIAN09 software to allow for peak assignment. Comparisons with literature have also been conducted to aid peak assignment [35–38].

## 3. Results and discussion

### 3.1. Device degradation study during burn-in period

**Figure 1.**
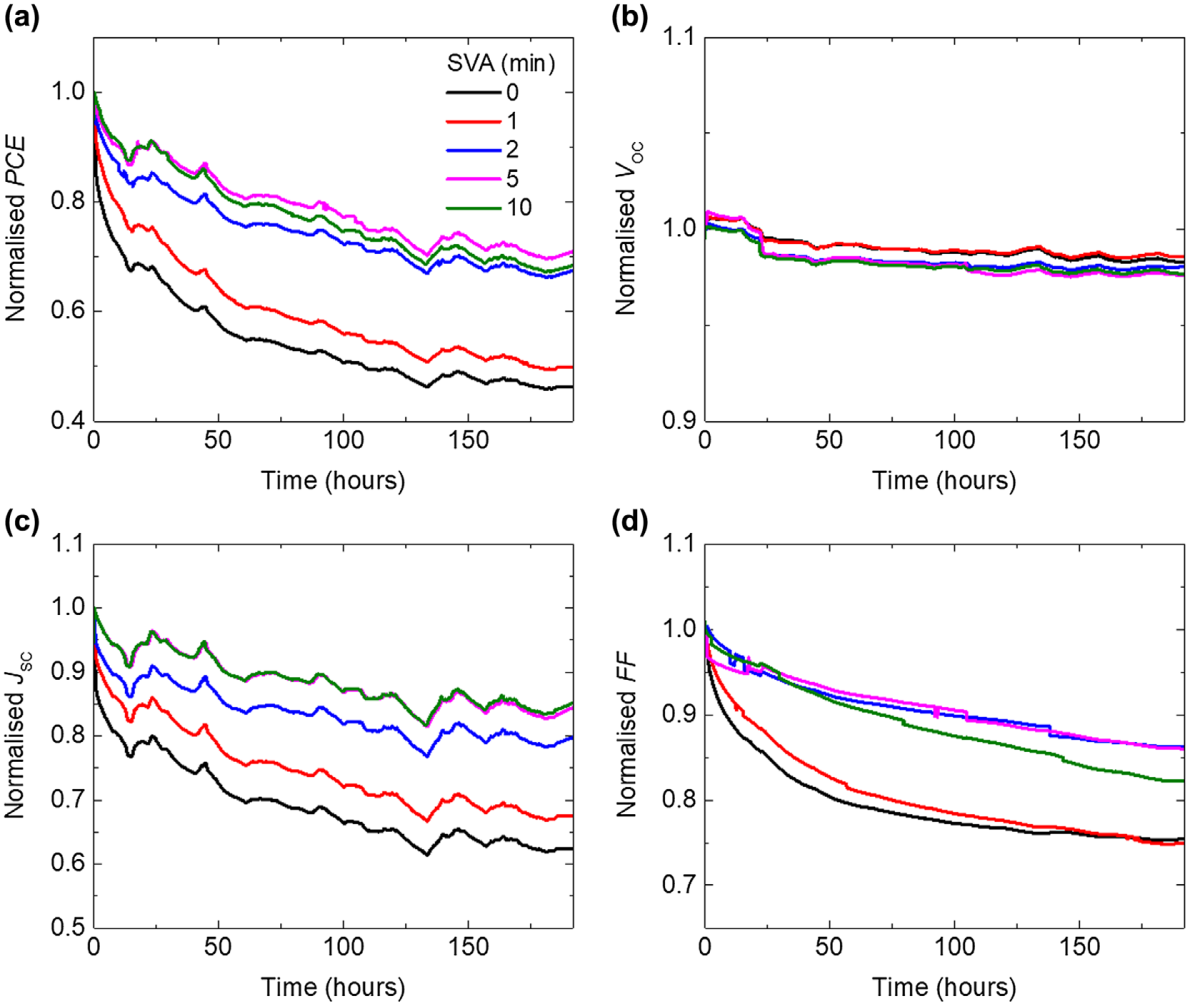
Normalised (a) *PCE*, (b) *V*_*OC*_, (c) *J*_*SC*_ and (d) *FF* (to the initial maximum values) of BTR:PC_71_BM devices, with active layers which have undergone increasing SVA time, as a function of photo-ageing time under one-sun equivalent illumination at room temperature in dry nitrogen.

Figure 1 shows the burn-in degradation of BTR:PC_71_BM devices as a function of SVA time. A moderate treatment time of 2 min is found to be optimal for the device performance, generating *PCE* of over 10%. Details of the fresh device performance are listed in Table 1. Long SVA times such as 5 and 10 min show slight reductions in the *PCE* probably due to high crystallinity of BTR phase as resolved by GI-XRD measurements, which are discussed in the next section. Overall, the longer the SVA, the less the burn-in is observed (Figure 1(a)). In particular, the *PCE* drops by 54.2% for 0 min SVA, 32.7% for 2 min SVA and 29.7% for 10 min SVA. The improvement in stability starts saturating for treatment longer than 2 min. Unlike a previous report which studies the stability of different small molecules with different crystallinity [10], here, we control the crystallinity of a material system via tuning the annealing time. Hence, the burn-in of the devices can be directly correlated to the crystallinity of the BTR:PC_71_BM blend films. The degradation curves of other cells in the same samples are available in Figure S1(a) and the degradation trends can be repeated using a separate batch of devices as shown in Figure S1(b).

**Table 1.** Performance of BTR:PC_71_BM devices with the active layers treated for increasing SVA time.

SVA time (min)	*J*_*SC*_ (mA/cm^2^)	*V*_*OC*_ (V)	*FF* (%)	*PCE* (%)
0	12.2	0.992	53.7	7.12
1	12.7	0.969	67.7	9.16
2	13.1	0.944	74.7	10.14
5	12.5	0.933	76.4	9.81
10	9.5	0.932	67.8	6.59

The reduced degradation for longer SVA is mainly due to a smaller drop in the short circuit current density (*J*_SC_) (Figure 1(c)). The drop in *FF* after 192 h degradation decreases with increasing SVA treatment time except the 10 min device (Figure 1(d)). Note that the open circuit voltage (*V*_*OC*_) is rather stable (Figure 1(b)) for all the devices implying that changes of the effective bandgap between the donor and acceptor are minor during degradation. Although the stability keeps improving with increasing SVA time, for practical use, we should also consider the absolute performance by taking the initial performance into account.

### 3.2. Crystallinity study of fresh and photo-aged films

**Figure 2.**
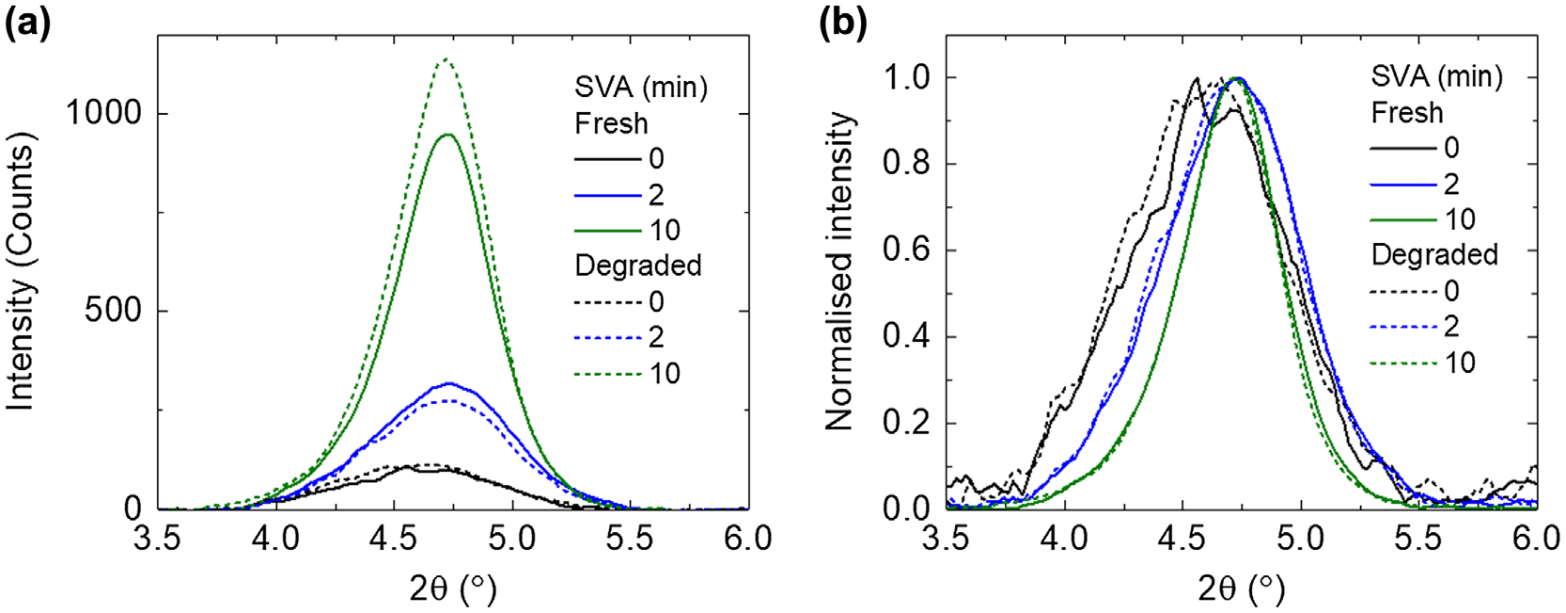
(a) GI-XRD spectra and (b) normalised GI-XRD spectra, of BTR:PC_71_BM films with increasing SVA time, before and after photo-ageing.

To probe if the crystallisation of the BTR phase was altered after photo-ageing, we performed GI-XRD on the BTR:PC_71_BM blend films before and after degradation. Three representative SVA times were selected, 0 min for the untreated, 2 min for the optimal, and 10 min for the over treated. As shown in Figure 2(a), there are diffraction peaks at 2*θ* = 4.72° for the treated films, corresponding to interlayer spacing of 1.87 nm which agrees with the published result [13]. The detailed changes in the peak positions are shown in Figure S2. Clearly, the absolute intensity of this diffraction peak increases with longer solvent treatment time. Furthermore, the full width at half maximum (FWHM) of the diffraction peak is reduced with increasing SVA time (Figure 2(b)): ~0.80° for 0 min SVA; ~0.68° for 2 min SVA; and ~0.49° for 10 min SVA. Both the increase in absolute intensity and reduction in FWHM show that the crystallisation increases with longer SVA time.

After the photo-ageing, there are only minor differences in the intensity between the diffraction peaks. Moreover, the FWHM of the diffraction peak is another way to probe the degree of crystallinity and, as shown in Figure 2(b), for all the fresh and photo-aged films, they are very similar in terms of the FWHM of the diffraction peak. These results suggest that photo-ageing of the blend films does not considerably affect the crystallinity or packing of the BTR.

### 3.3. UV–vis absorbance study of fresh and photo-aged films

**Figure 3.**
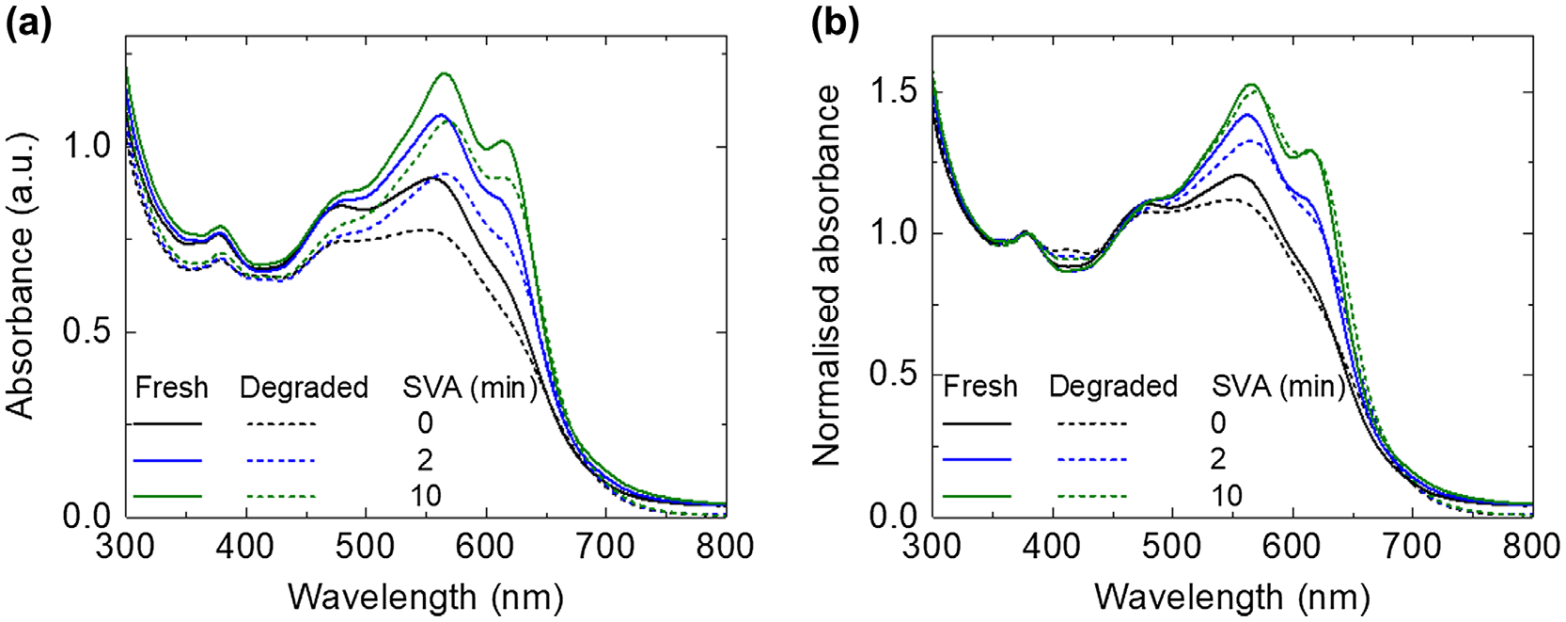
UV–vis absorbance spectra of the fresh and photo-aged BTR:PC_71_BM films: 0 min SVA, 2 min SVA and 10 min SVA films (a) without normalisation and (b) with normalisation to the PC_71_BM peak at 378 nm.

Photobleaching studies are commonly used to look at the degradation of organic materials [39,40]. UV–vis absorbance measurements were performed for the fresh and photo-aged films. For the fresh films, the intensity of the BTR peaks (~564 and ~614 nm) increases with longer SVA treatment, which confirms the crystalline/aggregate nature of these peaks. For all the films (SVA for 0 min, 2 min and 10 min), only slight photobleaching was observed (see Figure 3(a)), which could be linked to light induced degradation (photodissociation) [41]. The absorbance spectra also show that there is a small reduction in the absorbance at 378 nm (originating from PC_71_BM) for all the samples, suggesting PC_71_BM was degraded, and its degradation was found not to be affected by the SVA treatment. For better comparison to the change in the BTR absorption peaks (at 565 nm and 616 nm), all the spectra were normalised to the absorbance at 378 nm as shown in Figure 3(b). It is clear that after the photo-ageing, there are stronger reductions at the 565 nm peak compared with the 616 nm peak for all three blend films, which could be correlated to change in local molecular conformation (detailed discussion in the Raman section). The absorption spectra tend to show less photobleaching for longer SVA time (the main absorption peak drops by 15.2% for 0 min SVA; 14.6% for 2 min SVA; and 10.7% for 10 min SVA after photo-ageing).

### 3.4. Raman spectroscopy of fresh and photo-aged films

**Figure 4. F0004:**
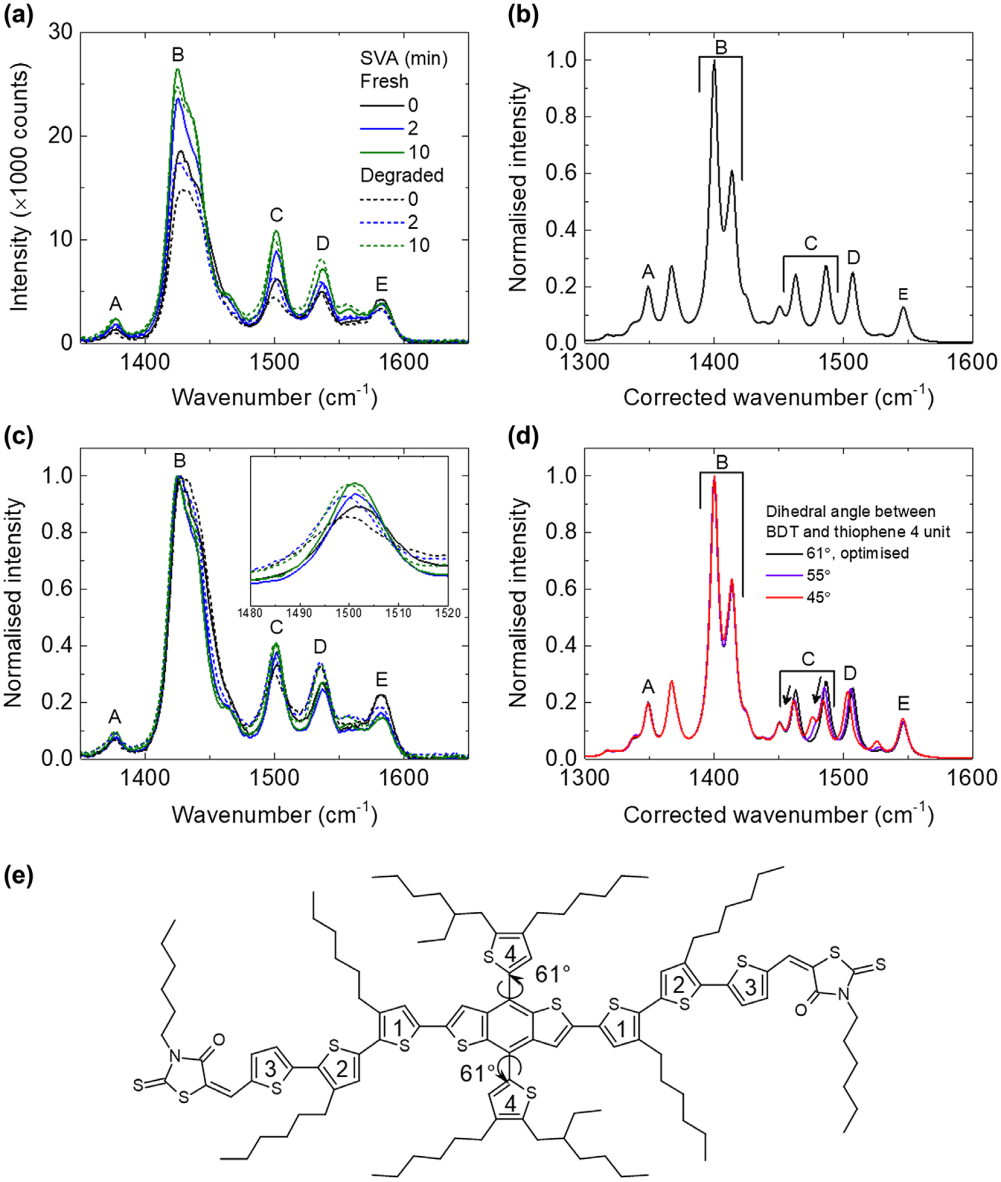
(a) Raman spectra of BTR:PC_71_BM films with increasing SVA time, before and after photo-ageing, (b) calculated Raman spectrum of BTR using B3LYP 6311G(d,p) with all alkyl side chains simplified to methyl groups, (c) normalised Raman spectra of BTR:PC_71_BM films with increasing SVA time, before and after photo-ageing (inset shows zoomed-in of peak C), (d) normalised calculated Raman spectrum of BTR with different dihedral angle between the BDT and thiophene 4 unit using the same simulation method and (e) the chemical structure of BTR with numbered thiophenes for Raman peak assignment. The main backbone has dihedral angles ranging from ~15° to 25°. The thiophenes numbered as 4 are ~61° out of the plane of the BDT core.

To study the effect of the photo-ageing to the molecular conformation of the blend films, Raman spectroscopy measurements were performed in which the change in Raman spectra could indicate a change in molecule conformation [35,36]. As shown in Figure 4(a), there are five peaks between 1350 and 1600 cm^−1^, and these peaks are all assigned to the BTR as PC_71_BM has negligible Raman signal in this region with this excitation condition.

Calculated Raman spectra at the B3LYP, 6-311G(d,p) level of theory were used to assign the Raman peaks of BTR (Figure 4(b) and Table 2). The calculated spectra showed a similar peak pattern and similar relative intensities to the experimental spectra but it was observed that after applying an empirical scaling factor of 0.9669 [34], the absolute frequency of the vibrational modes is underestimated. The peak at 1377 cm^−1^, corresponding to peak A, is assigned to the C-C stretching mode of the three backbone thiophene groups, labelled 1, 2 and 3 in the chemical structure in Figure 4(e). Peak B, corresponding to 1424 cm^−1^ is assigned to the C=C stretching of these backbone thiophene groups. The assignments of these two peaks are further supported by their similarity to the Raman spectra of P3HT [35,42,43]. Peak C, assigned to 1501 cm^−1^, has predominant contributions from the C=C stretching modes of thiophene 4 and the fused thiophenes in the benzodithiophene (BDT) unit which is consistent with several studies which assign peaks at a similar frequency to the BDT fused thiophene C=C modes [36,42,43]. In our simulations there are two peaks that could be assigned to peak C, but both clearly correspond to the vibrations above, the only difference between the two is the extent at which other vibrational modes of the molecule contribute. It should also be noted that vibrations in either the phenyl or thiophene part of the BDT unit will induce vibrations in the other, when assigning these peaks we have tried to discern the predominant stretch but that does not mean that this bond vibrates in isolation. Peak D, corresponding to 1537 cm^−1^, is assigned to the BDT unit, with the predominant vibration involving the un-fused phenyl bonds adjacent to the bonds with thiophene 4, again this assignment is consistent with the literature [36,42,43]. It is important to note that peak D is exclusively localised to the main conjugated backbone with clear on-axis vibrations of the core phenyl group, whilst peak C has a contribution from thiophene 4, which is perpendicular to the backbone. Peak E, assigned to 1582 cm^−1^_,_ is attributed to the BDT unit, with both the phenyl and fused thiophene C=C bonds showing strong vibrations, there is also a clear contribution from the C=C bonds of thiophene 1. The studies mentioned above [36,42,43], assign this mode to just the phenyl stretch of the BDT but our simulations show that this peak has clear contributions from the C=C bonds of both thiophene 1 and the fused thiophenes. This discrepancy may result from the different side chains of the BDT and thiophene units in the molecules studied.

**Table 2.** Assignments of the Raman peaks shown in Figure 4.

[Fig F0004]Peak	Vibrational mode
A	C-C stretching mode in ring of thiophene 1 and 2 (and 3 most likely) (like P3HT)
B	C=C of thiophene 1 and 2 and 3 (like P3HT)
C	C=C of fused thiophene and thiophene 4
D	BDT phenyl stretch (contribution from the fused thiophene – impossible for the phenyl ring to vibrate and the fused thiophene not vibrate as well) – (very tiny contributions from other thiophene)
E	Whole BDT unit, phenyl and fused thiophene, C=C in thiophene 1 and 2

The Raman intensity of the 1424 cm^−1^ peak from all the films decreases slightly after the photo-ageing, consistent with the slight photobleaching. Looking at the changes in absolute intensity is not a very reliable method to probe the degradation, as the intensity depends on sample-to-sample variation and film homogeneity. Therefore, the main Raman peak (peak B) is normalised and the relative intensity of the other peaks is studied. Here, we focus on the second main peak (peak C) at 1501 cm^−1^, as other peaks are too noisy to be probed accurately. We find that there is a trend where the relative intensity of the 1501 cm^−1^ peak (which also shifts to 1499 cm^−1^ after the photo-ageing) drops less with increasing SVA time (~10.5% for 0 min SVA, ~2.39% for 2 min SVA and ~1.46% for 10 min SVA) (see Figure 4(c)). The origin of the drop of 1501 cm^−1^ (peak C) relative to the 1424 cm^−1^ (peak B) maybe rationalised as follows: The optimised geometry shows a dihedral angle of 61° between the plane of thiophene 4 and the plane of the BDT unit (note that the dihedral angle is not quantitative and is used as a guide). The effect of changing this dihedral angle on the calculated Raman spectra is investigated as peak C is the only main peak with a considerable contribution from thiophene 4. It is important to note that this method does not provide us with a quantitative understanding but does allow us for understanding how relative changes to molecular conformation may result in changes to the Raman spectra. It is found that reducing the dihedral angle leads to a shift of peak C (both peaks assigned to peak C behave similarly) to lower wavenumbers and a reduction in the relative peak intensity with respect to the peak at 1424 cm^−1^ (Figure 4(d)), both correlate well with the experimental data. Therefore, it could suggest that degradation of BTR results from a molecular conformational change, that being a rotation of thiophene 4 towards the plane of the BDT. It suggests that increasing crystallisation (with longer SVA time) suppresses the rotation of thiophene 4 and thus improves the stability.

### 3.5. Photoluminescence study of fresh and photo-aged films

**Figure 5.**
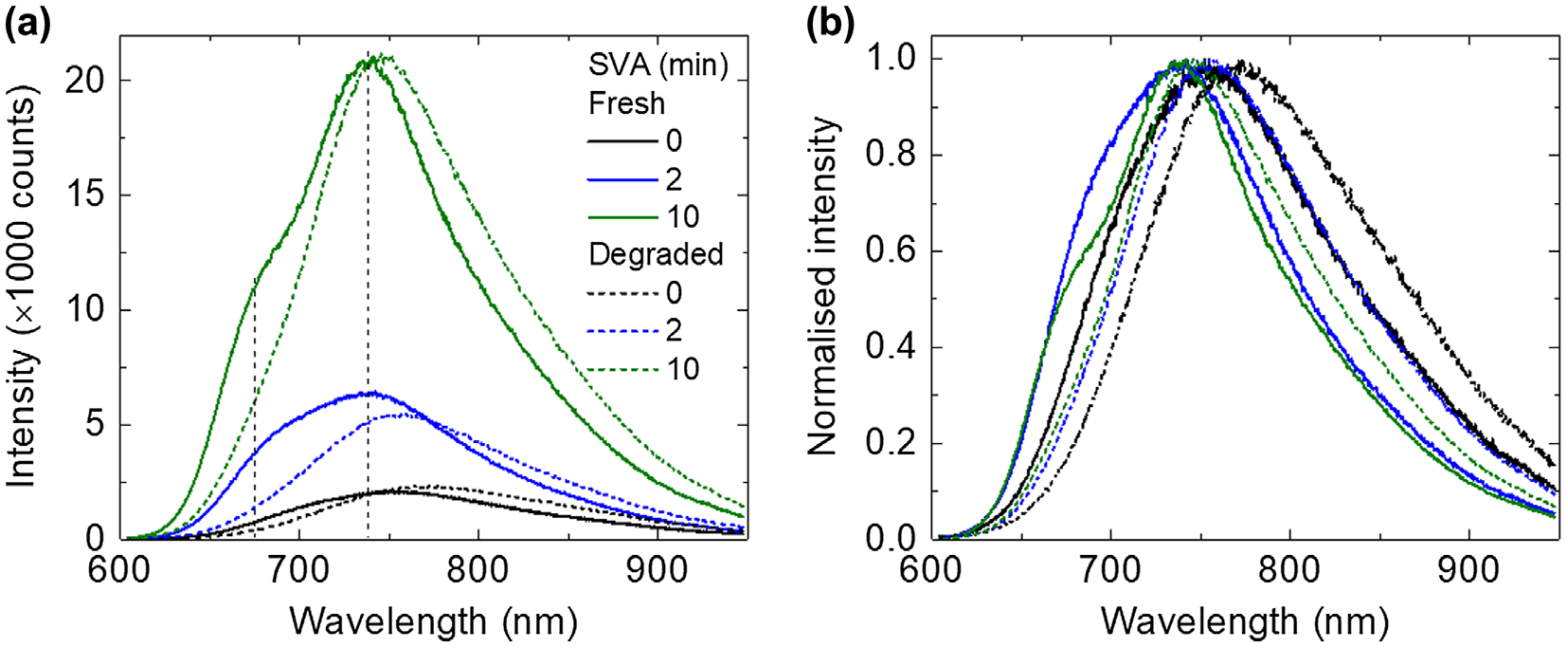
(a) PL spectra corrected by its absorbance at 532 nm (corresponding to the excitation wavelength) and (b) normalised PL spectra of BTR:PC_71_BM films with increasing SVA time before and after photo-ageing.

To gain further insights into the effect of photo-ageing to the photo-physical/chemical properties of the blend films, micro-PL measurements were performed at the same position for each sample as the corresponding Raman measurements for direct comparison. Since the absorption of the films corresponding to the excitation wavelength (532 nm) was changed after degradation, the PL spectra were scaled based on their absorption at 532 nm as shown in Figure 5(a). The original PL spectra are available in the Figure S3. The PL intensity of the main peak is relatively stable before and after ageing (variations are in the margin of error of PL and could be due to slight differences in focus from sample to sample). There is a shoulder at lower wavelength (680 nm) for the fresh samples that underwent SVA treatment. Both the PL main peak (740 nm) and the shoulder (680 nm) are in good correlation with the absorbance peaks of BTR, which could be ascribed to two transitions of the BTR crystalline phase. Moreover, the energy difference between the PL peaks is about 0.15 eV which is similar to the energy difference between the absorbance peaks of 0.18 eV. However, after ageing, the shoulder peaks at 680 nm vanish, consistent with the bigger drop in the lower wavelength absorbance peaks (564 nm).

The main PL peaks are normalised to show changes in peak position and spectral shape. As shown in Figure 5(b), after the photo-ageing, the PL peaks of all the films shift to longer wavelength (though it can also be due to change in relative intensity). The degree of shift of the peak position after photo-ageing tends to correlate with longer SVA (+ 22 nm for 0 min SVA, + 12 nm for 2 min SVA and + 8 nm for 10 min SVA). Overall, the change in PL spectra (both the disappearance of the shoulder peaks and the red-shift of the spectra after ageing) could be related to the change in molecular conformation, in particular the rotation of the thiophene 4 unit to the BDT core, affecting the photo-physical/chemical properties. The red-shift could be due to greater reduction in the PL intensity at the 680 nm shoulder peak than that at the 740 nm peak [44,45].

**Figure 6.**
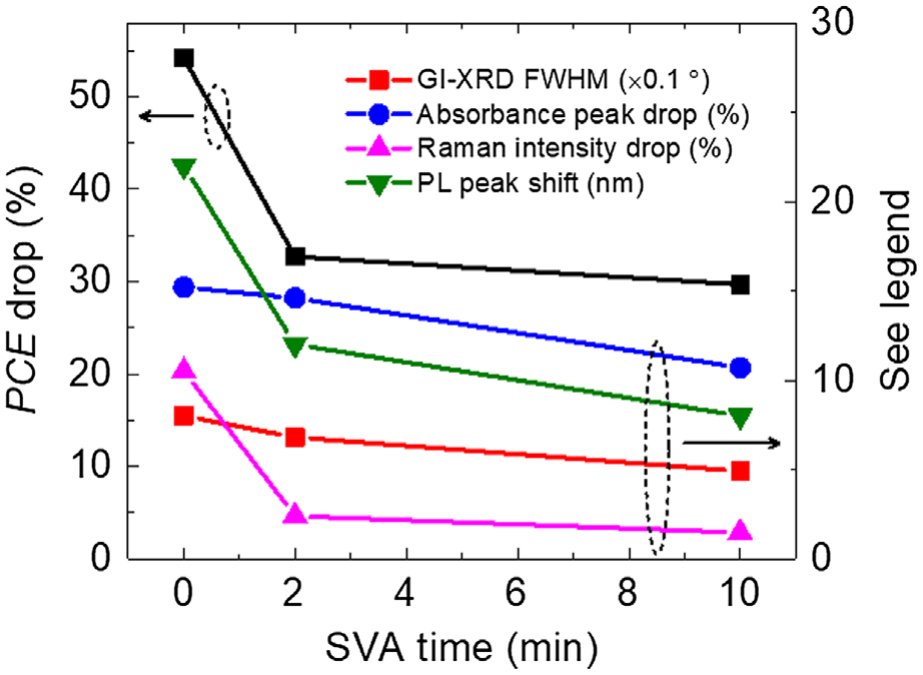
*PCE* drop, absorbance peak drop, relative Raman intensity drop and PL peak shift after the photo-ageing as a function of SVA time. For the GI-XRD FWHM, it is measured for fresh films (no obvious change after the photo-ageing).

Figure 6 summarises the drop in *PCE* (the burn-in region), the drop in the absorbance, the drop in the relative Raman intensity (1501 cm^−1^ to main peak) and the red-shift in PL peak position after the 192 h photo-ageing, as well as the FWHM of the GI-XRD diffraction peaks of the fresh blend films. Consistent correlations are observed between all the data. Such multi-characterisation allows us to compare between different techniques for the minor changes of the structural or optical properties causing the degradation. Here, our study mainly focuses on the role of the BTR donor, and further studies are needed to understand the role of PC_71_BM to the degradation. It is also worth noting that care must be taken when comparing photo-aged devices and blend films, as there are interlayers and electrodes in devices which may also affect the degradation even in a dry nitrogen environment, although we believe that the effect of interlayers and electrodes is likely mitigated under this inert environment.

## 4. Conclusions

We studied the photo-ageing effect of a highly efficient solution-processed SMSC system (BTR:PC_71_BM) as a function of SVA treatment time applied to the active layer under a dry nitrogen environment. We found that the reduction in burn-in with longer SVA time are correlated to the increase of crystallisation of the BTR phase in the blend films. The changes in UV–vis, Raman (experimental data and calculation), and PL are all consistent with a change in molecular conformation and the photo-physical/chemical change upon degradation. Since there is no clear difference in the GI-XRD diffraction peak after the photo-ageing, but clear difference and trend in the Raman spectra, we propose that the photo-ageing does not considerably affect the intermolecular packing of BTR, but does affect the local molecular conformation, in particular the reduction of the dihedral angles between the planes of the thiophene 4 and the BDT unit, supported by the change in the Raman (and PL) spectra after the photo-ageing. As the drop and difference in UV–vis absorbance of the photo-aged films cannot explain the significant drop and difference in the device efficiency, we suggest that the change in molecular conformation after the photo-ageing could lead to considerable change to electrical properties e.g. charge transport and recombination. Further work should be focused on the (photo-)electrical characterisation of the fresh and photo-aged devices. Overall, this work provides guide for the design of solution-processed small molecule donors from the viewpoint of their degradation mechanism, i.e. consideration of the side-chain rotation.

## Funding

This work was supported by EPSRC [grant number EP/M025020/1]; the Welsh Assembly Government funded Sêr Cymru Solar Project, the European Union’s Horizon 2020 research and innovation programme under the Marie Skłodowska-Curie [grant number 663830]; the National Research Network in Advanced Engineering Materials [grant number NRN093]; the Welsh Assembly Government Sêr Cymru II fellowship scheme, the National Natural Science Foundation of China [grant number 61674109]; the EPSRC Supergen SuperSolar Hub for an International and Industrial Engagement Award.

## Disclosure statement

No potential conflict of interest was reported by the authors.

## Supplemental data

Supplemental data for this article can be accessed here https://doi.org/10.1080/14686996.2018.1433948

## Supplementary Material

suppl.zip
