# Supplementary material for: Photo-stability study of a solution-processed small molecule solar cell system: correlation between molecular conformation and degradation
Source: Sci Technol Adv Mater. 2018 Feb 22;19(1):194–202. doi: 10.1080/14686996.2018.1433948 (PMC5827640; doi:10.1080/14686996.2018.1433948)
Supplement: suppl.zip [file TSTA_A_1433948_SM0165.zip › BTR stability SI_HL_Final.DOCX]

Supporting Information of

**Photo-stability study of a solution-processed small molecule solar cell system: correlation between molecular conformation and degradation**

Michael J. Newman^a^, Emily M. Speller^a^, Jérémy Barbé^a^, Joel Luke^b^, Meng Li^c^, Zhe Li^a^, Zhao-Kui Wang^c^, Sagar M. Jain^a^, Ji-Seon Kim^b^, Harrison Ka Hin Lee^a^, Wing Chung Tsoi^a^

^a^SPECIFIC, Department of Engineering, Swansea University, Swansea, UK

^b^Department of Physics and Centre for Plastic Electronics, Imperial College London, London SW7 2AZ, UK

^c^Institute of Functional Nano & Soft Materials (FUNSOM), Soochow University, 215123, China

Corresponding authors: Dr Harrison Ka Hin Lee and Dr Wing Chung Tsoi

Address: SPECIFIC, College of Engineering, Swansea University, Bay Campus, Fabian Way, Swansea SA1 8EN, United Kingdom

Emails: [K.H.Lee@Swansea.ac.uk](mailto:K.H.Lee@Swansea.ac.uk) and [W.C.Tsoi@Swansea.ac.uk](mailto:W.C.Tsoi@Swansea.ac.uk).


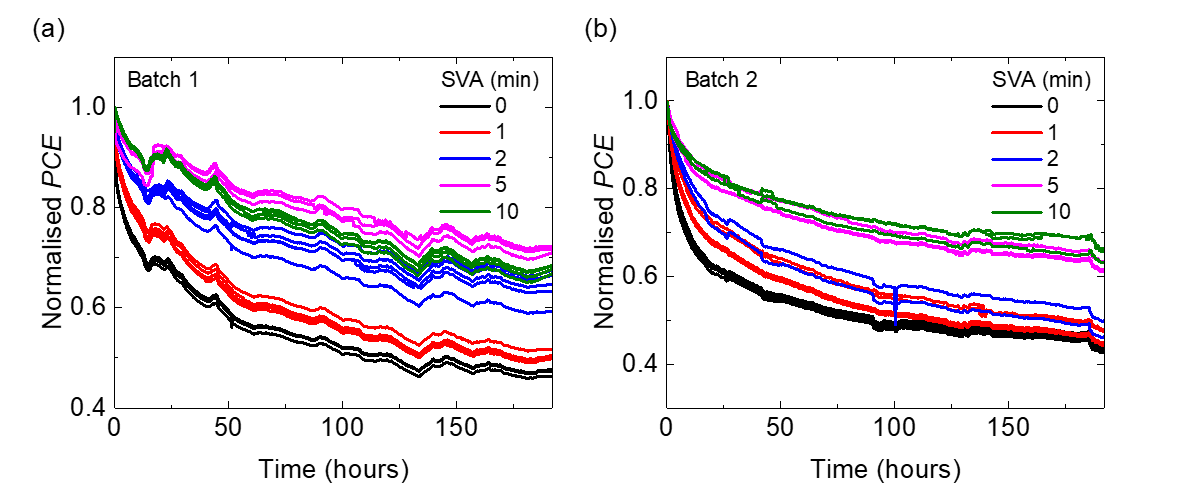


Fig. S1 Normalised *PCE* of devices in (a) batch 1 and (b) batch 2. Lines in the same colour are different cells within a sample.


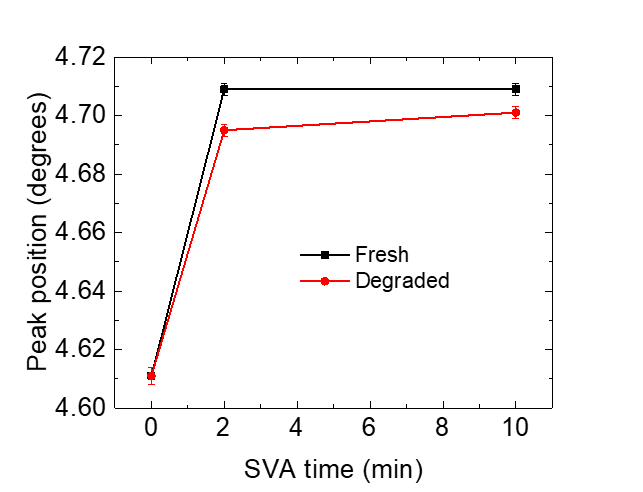


Fig. S2 GI-XRD peak positions of the BTR:PC_71_BM films with different SVA treatment times.


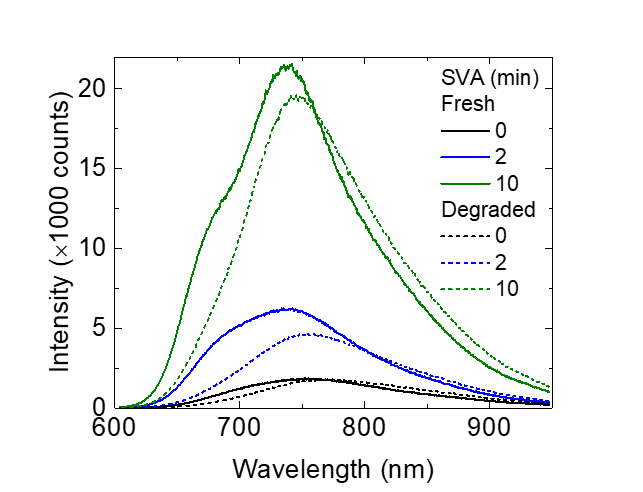


Fig. S3 PL spectra of BTR:PC_71_BM films with increasing SVA time, before and after photo-aging.
