# Supplementary material for: Photo-stability study of a solution-processed small molecule solar cell system: correlation between molecular conformation and degradation
Source: Sci Technol Adv Mater. 2018 Feb 22;19(1):194–202. doi: 10.1080/14686996.2018.1433948 (PMC5827640; doi:10.1080/14686996.2018.1433948)
Supplement: suppl.zip [file TSTA_A_1433948_SM0165.zip › suppl.pdf]

Supporting Information of

Photo-stability study of a solution-processed small molecule solar cell system: correlation between molecular conformation and degradation

Michael J. Newman[a], Emily M. Speller[a], Jérémy Barbé[a], Joel Luke[b], Meng Li[c], Zhe Li[a], Zhao-Kui Wang[c], Sagar M. Jain[a], Ji-Seon Kim[b], Harrison Ka Hin Lee[a], Wing Chung Tsoi[a]

[a]*SPECIFIC, Department of Engineering, Swansea University, Swansea, UK*

[b]*Department of Physics and Centre for Plastic Electronics, Imperial College London, London SW7 2AZ, UK*

[c]*Institute of Functional Nano & Soft Materials (FUNSOM), Soochow University, 215123, China*

Corresponding authors: Dr Harrison Ka Hin Lee and Dr Wing Chung Tsoi

Address: SPECIFIC, College of Engineering, Swansea University, Bay Campus, Fabian Way, Swansea SA1 8EN, United Kingdom

Emails: K.H.Lee@Swansea.ac.uk and W.C.Tsoi@Swansea.ac.uk.

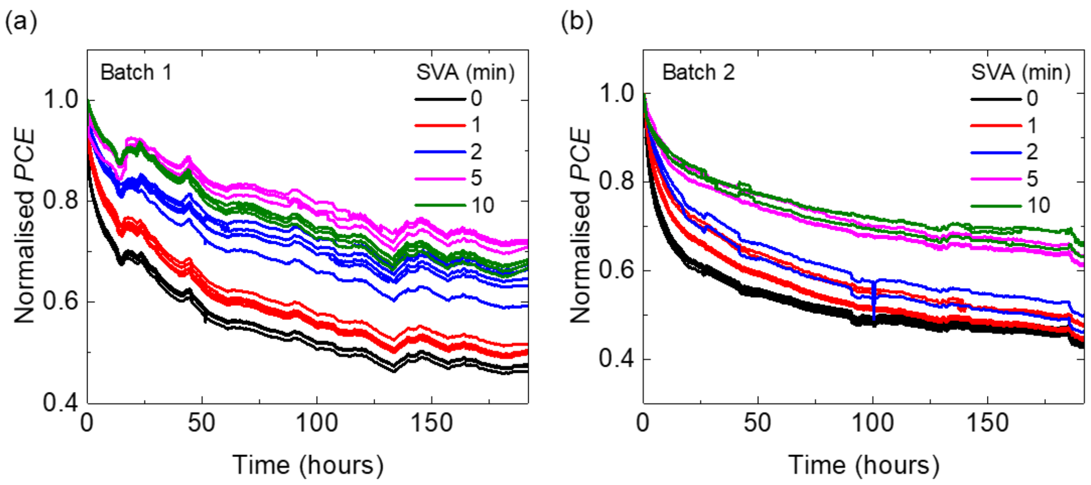

Fig. S1 Normalised *PCE* of devices in (a) batch 1 and (b) batch 2. Lines in the same colour are different cells within a sample.

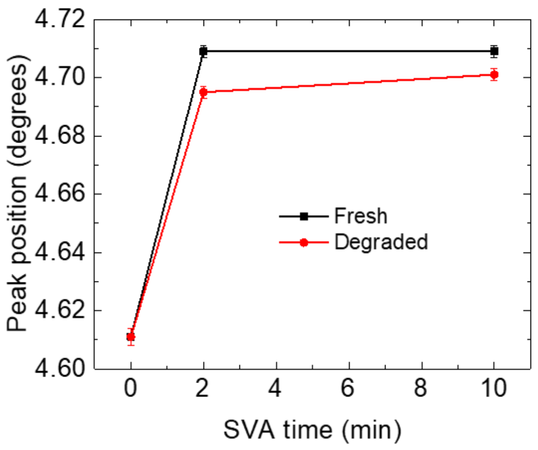

Fig. S2 GI-XRD peak positions of the BTR:$PC_{71}BM$ films with different SVA treatment times.

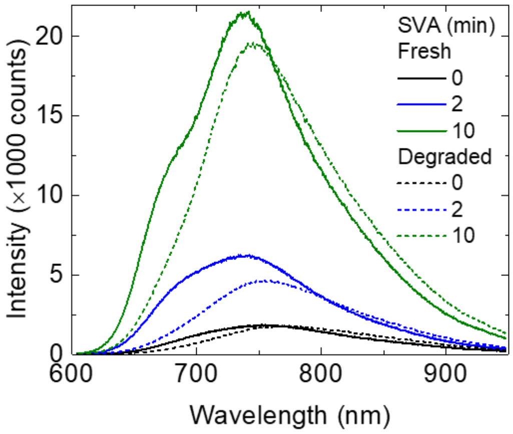

Fig. S3 PL spectra of BTR:$PC_{71}BM$ films with increasing SVA time, before and after photo-aging.
